# Central Precocious Puberty Caused by Novel Mutations in the Promoter and 5′-UTR Region of the Imprinted *MKRN3* Gene

**DOI:** 10.3389/fendo.2019.00677

**Published:** 2019-10-04

**Authors:** Pavlos Fanis, Nicos Skordis, Meropi Toumba, Nikoletta Papaioannou, Anestis Makris, Andreas Kyriakou, Vassos Neocleous, Leonidas A. Phylactou

**Affiliations:** ^1^Department of Molecular Genetics, Function and Therapy, The Cyprus Institute of Neurology and Genetics, Nicosia, Cyprus; ^2^Cyprus School of Molecular Medicine, Nicosia, Cyprus; ^3^Pediatric Endocrine Clinic, Paedi Center for Specialized Pediatrics, Nicosia, Cyprus; ^4^Department of Pediatrics, Iasis Hospital, Paphos, Cyprus; ^5^Developmental Endocrinology Research Group, School of Medicine, University of Glasgow, Glasgow, United Kingdom

**Keywords:** *MKRN3*, central precocious puberty (CPP), *MKRN3* promoter region, *MKRN3* 5′-UTR, gene mutations

## Abstract

**Background:** Central Precocious Puberty (CPP) is clinically defined by the development of secondary sexual characteristics before the age of 8 years in girls and 9 years in boys. To date, mutations in the coding region of *KISS1, KISS1R, PROKR2, DLK1*, and *MKRN3* genes have been reported as causative for CPP. This study investigated the presence of causative mutations in both the promoter and the 5′-UTR regions of the *MKRN3* gene.

**Methods:** Sanger DNA sequencing was used for screening the proximal promoter and 5′-UTR region of the *MKRN3* gene in a group of 73 index girls with CPP. Mutations identified were cloned in luciferase reporter gene vectors and transiently transfected in GN11 cells in order to check for changes in the activity of the *MKRN3* promoter. GN11 cells were previously checked for *Mkrn3* expression using lentivirus mediated knock-down. *In silico* analysis was implemented for the detection of changes in the mRNA secondary structure of the mutated *MKRN3* 5′-UTR.

**Results:** Three novel heterozygous mutations (−166, −865, −886 nt upstream to the transcription start site) located in the proximal promoter region of the *MKRN3* gene were identified in six non-related girls with CPP. Four of these girls shared the −865 mutation, one the −166, and another one the −886. A 5′-UTR (+13 nt downstream to the transcription start site) novel mutation was also identified in a girl with similar clinical phenotype. Gene reporter assay evaluated the identified promoter mutations and demonstrated a significant reduction of *MKRN3* promoter activity in transfected GN11 cells. *In silico* analysis for the mutated 5′-UTR predicted a significant change of the mRNA secondary structure. The minimum free energy (MFE) of the mutated 5′-UTR was higher when compared to the corresponding wild-type indicating less stable RNA secondary structure.

**Conclusion:** Our findings demonstrated novel genetic alterations in the promoter and 5′-UTR regulatory regions of the *MKRN3* gene. These changes add to another region to check for the etiology of CPP.

## Introduction

Central precocious puberty (CPP) is characterized by the premature activation of the hypothalamic-pituitary-gonadal axis due to the early activation of pulsatile Gonadotropin Releasing hormone (GnRH) secretion. Central precocious puberty is clinically defined by the development of secondary sexual characteristics before the age of 8 years in girls and 9 years in boys and is associated with a range of clinical and biological implications (1–3). The complex procedure of pubertal timing and progress are influenced by interactions of nutritional, environmental, socioeconomic, and genetic factors (4).

Strong evidence of the association of genetic factors on pubertal timing has been shown by population studies (5, 6). Using Genome Wide Association studies (GWAs) several genes have been associated with an increased growth and development, the regulation of the age at menarche, influence of energy homeostasis, and hormone regulation (7). The role of genetic determinants has been also illustrated by the similar age at menarche in mothers and daughters and among members of an ethnic group (8). Analysis among CPP patients has shown that 27.5% of cases are familial, thus suggesting an autosomal mode of inheritance (9). Although, the evidence suggests that age at the onset of puberty development is determined by genetic factors, the genetic etiology of CPP is largely unknown.

Several studies have used a candidate gene approach in an effort to identify genes associated with pubertal disorders. Currently, there is a steady increase in the number of genes associated with the development of hypogonadotropic hypogonadism and the Kallmann syndrome (10, 11). On the contrary only limited and rare molecular defects have been identified in individuals with CPP (12).

The genes that were discovered to be related with CPP and early GnRH secretion were the *KISS1*, its G protein-coupled receptor, *GPR54* (*KISS1R*), *PROKR2, DLK1*, and *MKRN3* (13–16). More precisely, the autosomal dominant *GPR54* mutation (p.Arg386Pro) was the first identified mutant that was proved to lead to prolonged activation of GnRH secretion through its ligand kisseptin (KISS1) (13). Another study that followed identified the p.Pro74Ser in the *KISS1* gene which is a defect that leads to the degradation resistance of kisspeptin and to the elevated availability of the protein (14). Therefore, these two gain-of-function *KISS1/KISS1R* mutations were the only causative mutations identified in CPP patients and that resulted to upregulation of the KISS1/KISS1R system leading to GnRH secretion and HPG activation (17). Similarly, a gain-of-function heterozygous mutation in the *PROKR2* (p.Cys242fsTer305) gene led to CPP by increasing the activity of the coexisting wild-type proteins (18). *DLK1* was the most recent gene in which genetic alterations were identified as a causal factor for CPP and in a recent report has also been associated with the age at menarche (8, 19). *DLK1* is maternally imprinted and its mutated allele follows the paternal mode of inheritance, such a case was a recent report with a large deletion of exon 1 in the *DLK1* gene (16). A second report followed and identified in female CPP patients three different frameshift mutations in the *DLK1*, also consistent with the maternal imprinting of the gene (20).

Nowadays, the most common genetic causes of CPP are the reported loss-of-function mutations in the *MKRN3* gene. Since the first ground breaking discovery of *MKRN3* gene, one of the major genes known to be causing CPP (15), various studies by other research groups identified a variety of other *MKNR3* loss-of-function mutations (21–27). *MKRN3* gene is located in the Prader-Willi syndrome (PWS)-related region (15q11-q13) on chromosome 15. The maternal allele of the gene is imprinted therefore *MKRN3* gene is expressed only from the paternal allele. All affected patients reported with familial CPP inherited the *MKRN3* mutations from their fathers (28).

*MKRN3* contains five zing finger domains: three C3H1 motifs, one C3HC4 RING motif, and one MKRN-specific Cys-His domain (29). C3H1 zinc-finger motifs are responsible for RNA binding while the RING motif is detected in E3 ubiquitin ligases and hence, it is assumed that has an ubiquitin-ligase activity (30). The MKRN3 protein is highly expressed in the developing brain including arcuate nucleus (15). It is believed that, MKRN3 is involved in protein degradation, therefore it might have an inhibitory effect in the pulsatile GnRH secretion (30). The exact mechanism that accomplish this effect or by which mechanism MKRN3 deficiency results in early reactivation of GnRH secretion is still unknown.

Although most of the reported studies describe loss-of-function mutations in the coding region of *MKRN3*, defects in the regulatory regions of the gene were described in two recent studies (19, 31). In the first study a four nucleotide deletion (c.-150_-147delTCAG) in the proximal promoter region of the *MKRN3* gene was found to be responsible for causing CPP (19). The second study describe a single nucleotide substitution at position 19 (*MKRN3:g*.+*19C*>*T*) from the transcription start site (TSS) in the 5′-UTR region of the *MKRN3* gene that is associated with CPP (31).

In this study, we report four novel heterozygous mutations located in the proximal promoter and 5′-UTR regions of the *MKRN3* gene in seven non-related girls with CPP. Functional analyses of the identified novel promoter/5′-UTR mutations indicated a robust correlation to the development of the disease.

## Materials and Methods

### Patients Under Study

The promoter/5′-UTR of the *MKRN3* gene was screened for variations in 73 index CPP girls referred to the Department of Molecular Genetics, Function and Therapy at the Cyprus Institute of Neurology and Genetics. Central precocious puberty in these girls was clinically evaluated by the development of secondary sexual characteristics before the age of 8 years, the basal and LHRH stimulated LH levels, advanced bone age, ovarian function on the pelvic ultrasound and normal central nervous system MRI. Mutations in the coding sequence of the *MKRN3, KISS1, KISS1R*, and *DLK1* genes were previously excluded. Written informed consent was obtained from parents of all patients under the age of 16 that participated in the study. The project was approved by the Cyprus National Ethics Committee and all methods were performed in accordance with the relevant guidelines and regulations.

### DNA Preparation

Genomic DNA was isolated from peripheral blood using the Gentra Puregene Kit (Qiagen, Valencia, CA, USA) according to the manufacturer's instructions. The DNA concentration and purity were measured using the Nanodrop ND-1000 spectrophotometer (NanoDrop Technologies, Wilmington, DE, USA).

### PCR Amplification and Sequencing

Variation analysis of the *MKRN3* proximal promoter and 5′-UTR region (−984 to +99 nucleotides (nt) from the TSS) was performed by PCR amplification followed by conventional Sanger DNA sequence analysis. The PCR reaction mixture was performed at a final volume of 20 μl and contained 2 μl AmpliTaq Gold PCR buffer I (10x), 2 μl dNTPs (2 mM), 0.5 μl of each primer (5 μM), 0.2 μl AmpliTaq Gold (0.5 U/μl) and 100 ng of genomic DNA. Amplification was performed with an initial denaturing temperature at 95°C for 10 min, followed by 35 cycles of denaturation (95°C, 30 s), annealing (58°C, 60 s), extension (72°C, 90 s), with a final extension at 72°C for 7 min. The following primers were used for PCR amplification; MKRN3_prom_F: 5′-TAACAGAATTCAGTATGCAGTCA−3′, MKRN3_prom_R: 5′-CGTGGGCTTCTGAGGGA−3′. The PCR products were cleaned-up with ExoSAP-IT reagent (Applied Biosystems, Foster City, CA) and sequenced using the same primers as for the PCR and the following internal primers; MKRN3_prom_int_F1: 5′-AGGGGACAGTGTCTTATTAG−3′, MKRN3_prom_int_F2: 5′-GAAGAGATTAAAGTAAAACC−3′ on a 3500xL Genetic Analyzer (Applied Biosystems, Foster City, CA).

### Vector Construction and Lentivirus Mediated Knock-Down

The wild-type and mutant proximal promoter/5′-UTR *MKRN3* sequences (−984_+99 nt relative to TSS) were amplified from human genomic DNA by PCR using the Phusion High-Fidelity DNA polymerase (New England Biolabs, Ipswich, MA, USA), cloned to pMIR_REPORT firefly luciferase reporter gene vector using KpnI and BamHI restriction sites (excision and replacement of the existing CMV promoter) and verified by sequencing.

For mouse *Mkrn3*, a clone from the TRC Mission (short hairpin) shRNA library (32) (Sigma Aldrich, St. Louis, MO, USA) was used for lentivirus mediated knockdown experiments in GN11 cells, including a scramble shRNA control clone (SHC002). GN11 cells were transduced with lentivirus and harvested 6 days later. Lentivirus was produced by transient transfection of 293T cells as described before (33, 34). The following clone was used from the TRC shRNA library: TRCN0000418150 (shMkrn3).

### Cell Culture and Transient Transfection

For the transfection studies we used the GnRH mouse neuronal GN11 cell line. GN11 cell line express very low levels of Gnrh1 mRNA and serve as cell culture model to study the rare population of hypothalamic GnRH neurons (35, 36). The GN11 cell line was kindly provided by Prof. Davide Lovisolo and Susanna Antoniotti (Department of Life Sciences and Systems Biology, University of Torino). GN11 cells were grown in Dulbecco's Modified Eagle Medium (DMEM; Invitrogen, Carlsbad, CA, USA) supplemented with 10% fetal bovine serum (FBS; Invitrogen, Carlsbad, CA, USA) and 1% Penicillin/Streptomycin (Invitrogen, Carlsbad, CA, USA) in a humid environment at 37°C with 5% CO_2_. Twenty four hours before transfection cells were plated in a 6-well plate at a density of 4 × 10^5^. The next day GN11 cells were transiently transfected with 500 ng/well of the pMIR_REPORT firefly luciferase vector together with 50 ng/well of the pGL4.74 *Renilla* luciferase vector (internal control) using the Lipofectamine 3000 Transfection Reagent (Invitrogen, Carlsbad, CA, USA). Twenty four hours later, total RNA from the GN11 transfected cells was isolated, cDNA was prepared and qPCR was performed (see *RNA extraction, Reverse Transcription (RT) and qPCR section*).

### RNA Extraction, Reverse Transcription (RT), and qPCR

Total RNA was isolated from GN11 cells with Trizol reagent (Invitrogen, Carlsbad, CA, USA). Before cDNA preparation the RNA was treated with DNaseI (Invitrogen, Carlsbad, CA, USA). cDNA was synthesized using the One *Taq* RT-PCR Kit (New England Biolabs, Ipswich, MA, USA) and amplified with the ABI 7900HT Fast Real-time PCR System (Applied Biosystems, New Jersey, USA). Each sample was amplified in triplicate. KAPA SYBR® FAST qPCR Master Mix (2X) Kit (Kapa Biosystems, Wilmington, MA, USA) was used to quantify the amplified products.

Relative expression analysis was performed as described before (37). Cycle threshold levels were calculated for each gene and normalized to values obtained for the endogenous house-keeping gene. For *Mkrn3* knock-down experiments the *18S* rRNA gene was used as a housekeeping gene and for the gene reporter assay the *Renilla luciferase* gene was used. The following primers were used for the *Mkrn3* knock-down experiments; 18S_RT_F: 5′-TGGTTGATCCTGCCAGTAG-3′, 18S_RT_R: 5′-CGACCAAAGGAACCATAACT-3′, Mkrn3_RT_F: 5′-TCCTGGACAGCCTTACCG-3′, Mkrn3_RT_R: 5′-TATGCACACCTGTCCCCAC-3′. For gene reporter assay the Fluc_RT_F: 5′-CTCACTGAGACTACATCAGC−3′ and Fluc_RT_R: 5′-TCCAGATCCACAACCTTCGC−3′ were used for the detection of firefly luciferase and the Rluc_RT_F: 5′-GAAGAGGGCGAGAAAATGGT−3′ and Rluc_RT_R: 5′-CCCTTCTCCTTGAATGGCTC−3′ were used for the detection of *Renilla* luciferase by qPCR.

### Western Blot Analysis

After protein transfer, the nitrocellulose membranes were blocked in 1% bovine serum albumin (BSA), incubated with the appropriate primary antibodies and developed using enhanced chemiluminescence. The membranes were probed with the following primary antibodies: Mkrn3 (ab177203), obtained from Abcam (Abcam, Cambridge, MA) and β-Actin (monoclonal AC-15, A1978) from Sigma (Sigma, St. Louis, MO, USA). HRP-conjugated IgG Goat Anti-mouse and Goat Anti-rabbit (Jackson ImmunoResearch, PA USA) secondary antibodies were used. The anti-MKRN3 antibody was used in 1:1,000 dilution and the anti-ACTIN antibody was used in 1:10,000 dilution in a solution of 1% non-fat dried milk in TBS/Tween. Secondary antibodies were used in a 1:20,000 dilution in non-fat dried milk in TBS/Tween. The levels of *Mkrn3* protein were normalized with the corresponding β-Actin loading control within the same immunoblot. Visualization of the immunoblots and acquisition of the images was carried out the UVP BioSpectrum Imaging System (UVP, LLC, Upland, CA, USA). Dynamic Total Time Exposure settings were used, with binning set at 12.1 MP, 149% and 1 × 1 interpolation. Exposure times varied depending on the primary antibody used.

### RNA Secondary Structure Analysis

The 99 nt 5′-UTR mRNA of *MKRN3*, was submitted to the RNAfold WebServer (http://rna.tbi.univie.ac.at/cgi-bin/RNAWebSuite/RNAfold.cgi) with default parameters to predict the potential secondary structure. Secondary structures were predicted for the wild-type and the *MKRN3:g*.+*13C*>*T* mutant mRNA. Potential minimum free energy (MFE) structures, centroid structures, and positional entropies were obtained (38).

### Statistical Analysis

Statistical analysis was performed with the GraphPad Prism 8 software using two tailed unpaired *t*-test with confidence level of 95%. *P* < 0.05 were considered significant.

## Results

### Identification of Novel *MKRN3* Promoter/5′-UTR Mutations

**Figure 1:**
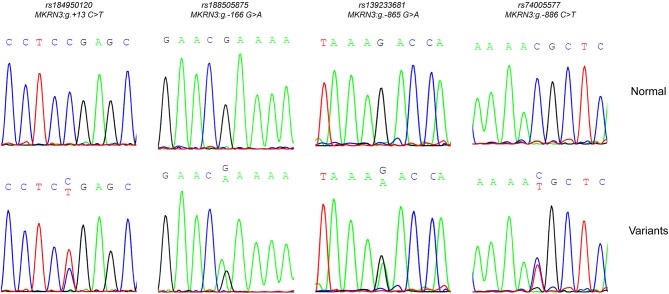
DNA sequencing analysis. Part of the sequencing electropherograms of the *MKRN3* proximal promoter showing the novel heterozygous mutations identified; *rs184950120 (MKRN3:g*.+*13C*>*T), rs188505875 (MKRN3:g.-166G*>*A), rs139233681 (MKRN3:g.-865G*>*A), and rs74005577 (MKRN3:g.-886C*>*T)*. For each mutation the corresponding normal sequencing electropherograms is showed.

Sequencing analysis of the promoter and 5′-UTR regions of the *MKRN3* gene in the cohort of 73 index girls with CPP revealed three novel heterozygous mutations (−166, −865, −886 nt upstream to the TSS) located in the proximal promoter region and one novel heterozygous mutation located in the 5′-UTR (+13 nt downstream to the TSS) region (Figure 1). Three of these girls shared the promoter *MKRN3* mutations*: g.-865G*>*A, g.-166G*>*A* and *g.-886C*>*T*. A fourth 7.6 years old girl at the time of diagnosis was identified with the 5′-UTR *MKRN3:g*.+*13C*>*T* mutation. All identified mutations were traced in dbSNP with the following reference SNP ID numbers: rs184950120 (*MKRN3:g*.+*13C*>*T*), rs188505875 (*MKRN3:g.-166G*>*A*), rs139233681 (*MKRN3:g.-865G*>*A*), and rs74005577 (*MKRN3:g.-886C*>*T*) but they have never been linked with CPP. The allele frequencies of the identified mutations in different population studies are described in Table 1. In addition the four mutations were absent in samples from 50 individuals that were randomly selected from the general Cypriot population. Investigation by DNA sequencing of both parents for two of the CPP girls bearing the *MKRN3:g.-865G*>*A* mutation and one bearing the *MKRN3:g*.+*13C*>*T* revealed the presence of the novel mutations only in the fathers. DNA from the parents of the remaining three CPP girls was not available. Since the *MKRN3* gene is maternally imprinted with only the paternal allele expressed, these findings were in agreement with the paternal mode of inheritance for the *MKRN3* mutations (15).

**Table 1:** Allele frequencies of the identified mutations in different population studies.

**Study**	**Population**	**Group**	**Sample size** **(allele number)**	**Ref allele** **(allele number)**	**Alt allele** **(allele number)**
**rs184950120 (*****MKRN3:g.+13C>T)***					
TopMed	Global	Study-wide	125,568	*C* = 0.99710 (125,205)	*T* = 0.00289 (363)
gnomAD—Genomes	Global	Study-wide	30,942	*C* = 0.9948 (30,780)	*T* = 0.0052 (162)
1000Genomes	Global	Study-wide	5,008	*C* = 0.998 (4,999)	*T* = 0.002 (9)
**rs188505875 (*****MKRN3:g.-166G>A*****)**					
gnomAD—Genomes	Global	Study-wide	30,803	*G* = 0.993 (30,577)	*A* = 0.007 (226)
1000Genomes	Global	Study-wide	5,008	*G* = 0.999 (5,001)	*A* = 0.001 (7)
**rs139233681 (*****MKRN3:g.-865G>A*****)**					
TopMed	Global	Study-wide	125,568	*G* = 0.99501 (124,942)	*A* = 0.00499 (626)
gnomAD - Genomes	Global	Study-wide	30,976	*G* = 0.9951 (30,825)	*A* = 0.0049 (151)
1000Genomes	Global	Study-wide	5,008	*G* = 0.992 (4,969)	*A* = 0.008 (39)
**rs74005577 (*****MKRN3:g.-886C>T*****)**					
TopMed	Global	Study-wide	125,568	*C* = 0.99314 (124,707)	*T* = 0.00686 (861)
gnomAD—Genomes	Global	Study-wide	30,972	*C* = 0.9943 (30,797)	*T* = 0.0057 (175)
1000Genomes	Global	Study-wide	5,008	*C* = 0.994 (4977)	*T* = 0.006 (31)

### Evaluation of the Identified Novel Mutations in the Promoter/5′-UTR Region of the *MKRN3* Gene

Luciferase reporter assay was employed to investigate the hypothesis that the identified novel *MKRN3* mutations were the functional cause of the CPP. The *MKRN3* promoter/5′-UTR containing either the wild-type or the different mutations was isolated and cloned into a luciferase gene reporter vector. Promoter/5′-UTR constructs containing the wild-type, the *MKRN3:g*.+*13C*>*T*, the *MKRN3:g.-166G*>*A*, the *MKRN3:g.-865G*>*A*, or the *MKRN3:g.-886C*>*T* mutations were designed and tested for activity in GN11 cells. GN11 cells express low levels of Gnrh1 mRNA and maintain many of the phenotypic characteristics of immature GnRH neurons *in vitro* therefore, serve as cell culture model to study the rare population of hypothalamic GnRH neurons (35, 39).

**Figure 2:**
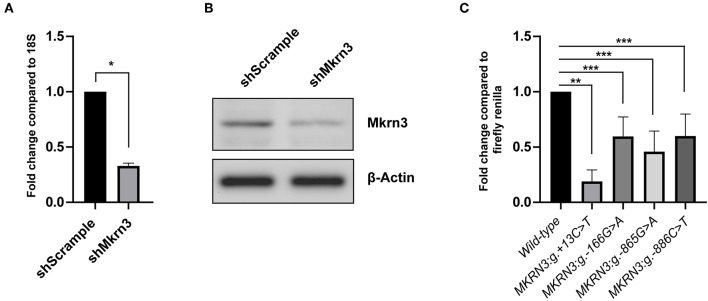
**(A,B)** shRNA knockdown efficiency on **(A)** RNA and **(B)** protein *Mkrn3* levels. GnRH expressing GN11 cells were treated with the indicated shRNA. **(A)** Total mRNA analyzed by RT-qPCR. Relative expression of *Mkrn3* was analyzed with respect to the *18S* gene. **(B)** Whole cell lysates were analyzed by western blotting with Mkrn3 antibody. The β-Actin stained membrane serves as loading control. The sh*Mkrn3* reduces the mRNA and protein levels of Mkrn3. As a negative control a shRNA containing a scrambled sequence was used (shScrample). **(C)** The novel *MKRN3* promoter/5′-UTR mutations reduce the promoter activity in GN11 cells. The *MKRN3* promoter reporter gene constructs (spanning nucleotides −984_+99 relative to TSS) containing the MKRN3:g.+13C>T, *MKRN3:g.-166G*>*A, MKRN3:g.-865G*>*A*, and *MKRN3:g.-886C*>*T* mutations were transiently transfected in GN11 cells. Luciferase activities were calculated relative to the wild-type *MKRN3* promoter reporter construct. Results are the average of three independent experiments with each sample assayed in triplicate. *P < *0.0001;* **P < *0.001;* ***P < *0.05*.

GN11 cells were first tested if they are a suitable model to perform the gene reporter assay. Thus, protein extracts were isolated, loaded on an SDS-PAGE gel and tested for endogenous Mkrn3 expression by staining with an anti-Mkrn3 antibody. Since there was lack of information for the specificity of the antibody we perform knock-down experiment of the endogenous *Mkrn3* using shRNA. We used a shRNA against *Mkrn3* (shMkrn3) that reduced the mRNA and protein levels of *Mkrn3* indicating the presence of *Mkrn3* expression in GN11 cells (Figures 2A,B).

The novel promoter *MKRN3:g.-166G*>*A, MKRN3:g.-865G*>*A*, and *MKRN3:g.-886C*>*T* mutations were found to significantly decrease the transcription of the luciferase gene in GN11 cells by ~0.4 fold relative to the wild-type (Figure 2C). The strongest effect with ~0.8 fold decrease in the transcription of the luciferase gene was observed with the novel *MKRN3:g*.+*13C*>*T* mutation which is located in the 5′-UTR region of the *MKRN3* gene (Figure 2C, second column bar).

Complete clinical characteristics were available for six out of seven of these cases (Table 2). The median age at puberty onset of these patients was 6.55 years and the median age at referral was 8.05 years. These patients displayed the typical characteristics of precocious puberty (Table 2).

**Table 2:** Clinical and laboratory characteristics for six girls with *MKRN3* mutations.

**Proband**	***MKRN3* mutation**	**Age at onset (years)**	**Age at referral (first visit) (years)**	**Stage of breast development**	**Stage of pubic hair**	**Stage of axillary hair**	**Bone age (years)**	**LHRH test FSH/LH(U/L)**			**MRI**	**Pelvic ultrasound**	**Comments/Other symptoms**
								**0**	**30**	**60**			
1	MKRN3:g.-865G>A	5.4	7.4	B3	P2	A1	10	1.95/5.3	8.9/9	7.7/7	No	Pubertal	Hearing impairment/cochlear implants/first cousin from father's side same clinical picture
2	MKRN3:g.-865G>A	7	7.8	B4	P3	A1	12	3.2/2.4	14/19	12/21	Normal	Pubertal	
3	MKRN3:g.-865G>A	N/A	9.5	B5	P4	A2	11.5	5.1/4.8	–	–	No	Pubertal	Patient came at age 9.5 years with menarche
4	MKRN3:g.-865G>A	6.1	8.8	B3	P2	A2	9.6	3/4	15/19	16/15	No	Normal	
5	MKRN3:g.-886C>T	N/A	8.3	B4	P4	A4	N/A	3/3	5/12	4/9	Normal	Normal	Ovarian volume: post pubertal
6	MKRN3:g.+13C>T	7.6	7.6	B2	P2	A1	8.5	0.25/3.5	–	–	No	No	Patient came back at age 9.1 y with menarche/Obesity-insulin resistance

The affected girl bearing the novel *MKRN3:g*.+*13C*>*T* mutation was referred due to premature sexual maturation at the age of 7.5 years. She was at Tanner stage 2 of puberty with elevated gonadotrophins, bone age advancement for 2 years compared to chronological age while a pelvic ultrasound showed ovarian function. She was treated with GnRH analogs to suppress her pubertal progression with the aim to increase her final height potential and to alleviate any psychological issues. Additionally she was obese with body mass index (BMI) more than +2SDS for her age and gender and of course her height was taller than her midparental target height. During her 2 years of follow up with CPP treatment her weight increased further and she showed signs of insulin resistance for which she was commenced on treatment with metformin.

These results demonstrate that the *MKRN3* promoter and especially the 5′-UTR novel mutations have a negative effect on gene regulation.

### *In silico* Analyses of the Novel *MKRN3:g.+13C>T* 5′-UTR Mutation

**Figure 3:**
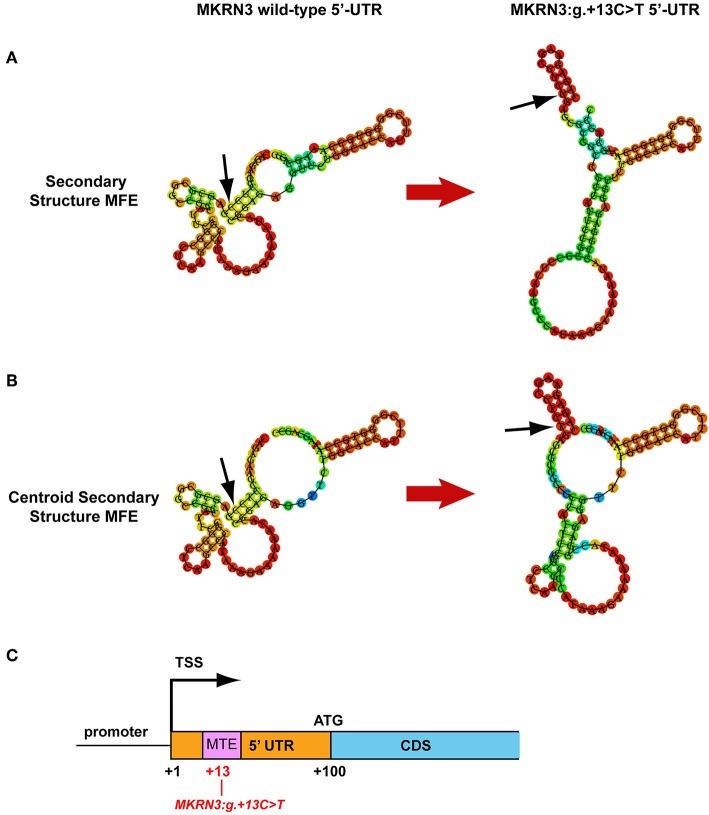
*MKRN3* 5′-UTR *in silico* predictions. **(A,B)** Predicted *MKRN3* 5′-UTR mRNA secondary structures showing the MFE and centroid MFE secondary structure, respectively, for the wild- type and *MKRN3:g*.+*13C*>*T* mutant. **(C)** Position of the Motif Ten Element (MTE) in relation with the *MKRN3:g*.+*13C*>*T* mutation. Black arrows indicate the position of the *MKRN3:g*.+*13* nucleotide.

Since the luciferase reporter assay of the novel 5′-UTR mutation indicated a strong effect in the reduction of the transcriptional activity of the gene we further investigated this by performing *an in silico* mRNA secondary MFE structure prediction. Secondary MFE structure of the wild-type *MKRN3* 5′-UTR mRNA was compared to the secondary MFE structure of the *MKRN3:g*.+*13C*>*T* 5′-UTR mutant mRNA. Figures 3A,B shows the predicted MFE and centroid MFE secondary structures of the *MKRN3* 5′-UTR mRNA. The effect of the mutated nucleotide is visible as it changes the MFE secondary structures of the *MKRN3:g*.+*13C*>*T* mutant compared to the wild-type 5′-UTR mRNA. Moreover, the centroid secondary structure MFE values of the predicted mRNA secondary structures were also found to be higher in the *MKRN3:g*.+*13C*>*T* mutant when compared to the wild-type form (Table 3). This finding could be attributed to a probable reduced stability of the secondary mRNA structure.

**Table 3:** Minimum free energy (MFE) and centroid secondary structure MFE values.

	**Secondary structure MFE (kcal/mol)**	**Centroid secondary structure MFE (kcal/mol)**	**Free energy of the thermodynamic ensemble**	**Frequency of the MFE structure %**	**Ensemble diversity**
Wild type	−29.70	−27.60	−30.80	16.70	18.94
*MKRN3:g.+13C>T* (5′-UTR)	−32.10	−20.50	−33.47	10.88	19.76

*mRNA secondary structures values with 1 kcal/mol above the minimum MFE observed in wild type are considered responsible for the destabilization of the structure (40)*.

Furthermore, using the MatInspector tool of Genomatix software suit we performed *in silico* analysis of the 5′-UTR genomic region for alterations in binding sites downstream of the TSS. Comparison between the wild-type and mutated sequence predicted that the novel 5′UTR *MKRN3:g*.+*13C*>*T* mutation would lead to the loss of a putative Motif Ten Element (MTE) binding site that can promote transcription by RNA polymerase II (41) (Figure 3C).

## Discussion

Mutations in the intronless *MKRN3* gene can cause CPP, a gonadotropin-dependent precocious puberty due to the early activation of the hypothalamic-pituitary-gonadal axis (2). To date, ~40 different mutations in the *MKRN3* gene have been reported and associated with CPP (21–25, 42). *MKRN3* gene is an imprinted gene expressed only from the paternal allele; therefore affected patients with familial CPP inherited the *MKRN3* mutations from their fathers. Most of the published studies describe causative mutations in the coding region of *MKRN3* gene with only two recent studies report defects in the regulatory regions of the gene (19, 31). In the first study a CPP causative small deletion (c.-150_-147delTCAG) in the promoter region of the *MKRN3* gene was identified (19). The second study described a CPP causative mutation (MKRN3:g.+19C>T) located in the 5′-UTR region of the *MKRN3* gene (31).

In this study we identified three novel heterozygous mutations located in the proximal promoter and one in the 5′-UTR region of the *MKRN3* gene in a total number of seven non-related girls with CPP. Four of these girls shared the *MKRN3:g.-865G*>*A* mutation, one the *MKRN3:g.-166G*>*A* and another one the *MKRN3:g.-886C*>*T* mutation, all located in the proximal promoter of the gene. Interestingly, a 7.6 years old girl with CPP at the time of diagnosis was identified with the novel *MKRN3:g*.+*13C*>*T* mutation in the 5′-UTR region. A large study involving ~370,000 women also identified the *MKRN3:g*.+*13C*>*T* 5′-UTR mutation as a putative marker of puberty timing when paternally inherited (43).

**Figure 4:**
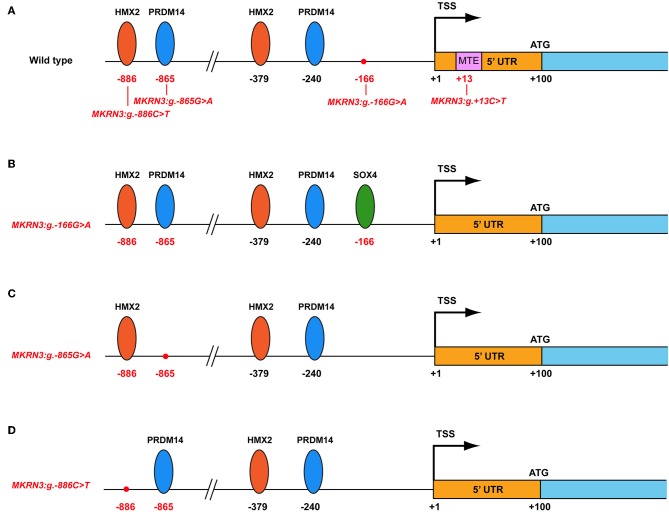
Schematic representation of the *MKRN3* gene with various predicted transcription factors. The various transcription factors are indicated with oval shapes. The positions of the mutations identified are indicated in relation with TSS. **(A)** Wild-type *MKRN3* promoter region. **(B)** *MKRN3* promoter region with the novel *MKRN3:g.-166G*>*A* mutation. **(C)** *MKRN3* promoter region with the novel *MKRN3:g.-865G*>*A* mutation and **(D)** *MKRN3* promoter region with the novel *MKRN3:g.-886C*>*T* mutation. TSS: transcription start site; HMX2: H6 Family Homeobox 2; PRDM14: PR/SET Domain 14; SOX4: SRY-Box 4.

*In silico* analysis using the MatInspector tool for the identification of possible changes in transcription factor (TF) binding sites in the proximal promoter of wild-type and mutated *MKRN3* gene predicted various modifications (Figure 4). Specifically, when the *MKRN3:g.-166G*>*A* is present, a putative binding site for the SOX4 is created (Figure 4B). SOX4 is a transcription factor involved in the regulation of embryonic development and in the determination of the cell fate. Additionally, this same transcription factor is mainly expressed in neural cells that have already been dedicated to neuronal differentiation (44, 45) and is highly expressed in the majority of hypothalamic GnRH neurons in adult mice (46). We postulate that with the creation of its binding site, SOX4 is recruited to the promoter of *MKRN3* and act as a repressor that would lead to decrease of the expression of *MKRN3*. The *MKRN3:g.-865G*>*A* mutation lead to loss of the PRDM14 transcription factor binding site (Figure 4C). *MKRN3* promoter is predicted to have two binding sites for the PRDM14 at positions −240 and −865 relative to TSS (Figure 4). PRDM14 recruits PRMT5 to mediate histone arginine methylation and control neural stem cell differentiation (47). In this study we hypothesize that loss of one of the two PRDM14 binding sites in the *MKRN3* promoter might lead to reduction in *MKRN3* expression. Similarly, *MKRN3:g.-886C*>*T* mutation lead to loss of one out of two HMX2 transcription factor binding sites in the *MKRN3* promoter (Figure 4D); therefore reduction in *MKRN3* expression. HMX2 is a transcription factor which is expressed in the developing hypothalamus and especially in the growth hormone-releasing hormone (GHRH) neurons of the arcuate nucleus (48). Gene reporter assay studies in GN11 cells with vectors containing the different mutated promoters' revealed significant reduction in the *MKRN3* promoter activity, suggesting that the mutations identified in the promoter region had a negative effect on *MKRN3* transcription.

The novel *MKRN3:g*.+*13C*>*T* mutation located in the 5′-UTR had the strongest effect with the gene reporter assay. The fact that secondary structures often occur within 5′-UTR with followed impact in the regulation of translation (49), lead us to perform mRNA secondary MFE structure prediction. Comparison of the *MKRN3* wild-type and the *MKRN3:g*.+*13C*>*T* mutated 5′-UTR mRNA secondary structures showed distinct differences. The higher centroid secondary structure MFE values of the mutant mRNA indicate less stable mRNA secondary structure. Moreover, using the MatInspector prediction tool the core promoter transcription factor binding site MTE located in 5′-UTR region is lost in the presence of the *MKRN3:g*.+*13C*>*T* mutation indicating a negative impact on *MKRN3* transcription.

Although genetic analysis of both parents for only two of the CPP girls bearing the *MKRN3:g.-865G*>*A* and the *MKRN3:g*.+*13C*>*T* mutations confirmed the paternal mode of inheritance for the *MKRN3* mutations, it is hoped in future genetic analyses will be performed also for the parents of the rest of CPP girls described in this study. Finally, as mutations in the promoter and 5′-UTR region of the *MKRN3* gene are present and cause CPP, we cannot exclude the possibility of the presence of mutations in the promoter region of other CPP related genes for these (and other) CPP patients.

In summary, our current findings suggest that novel mutations in the *MKRN3* proximal promoter and especially in the 5′-UTR region resulted in *MKRN3* deficiency therefore contributing to the clinical manifestation of CPP phenotype.

## Data Availability Statement

The raw data supporting the conclusions of this manuscript will be made available by the authors, without undue reservation, to any qualified researcher.

## Author Contributions

All authors listed have made a substantial, direct and intellectual contribution to the work, and approved it for publication.

### Conflict of Interest

The authors declare that the research was conducted in the absence of any commercial or financial relationships that could be construed as a potential conflict of interest.
